# The Partially Insane

**Published:** 1907-10

**Authors:** 


### The Partially Insane.

It has been said that the sane majority put the insane minority in asylums just as in politics the majority puts the minority out of office. It has been surmised that as the insane minority is increasing so rapidly they will, in time, constitute the majority and put the sane minority in the asylums for acting queer, just as in politics when the minority grows till it becomes the majority puts the former majority in seclusion. We have, however, a very large class of half-crazy people who in politics would be designated as "on the fence." In view of this semipsychic weakness the community in which these semi-unfortunates live owes to them the necessary care and treatment, and to itself measures to protect itself from their misdeeds. Here we find ourselves right in the midst of the question of partial responsibility. It is unjust to punish the partially insane as though he were entirely sane, and it is quite as unfair to restrain him as though he were actually insane. Grassett, of Montpelier, in a very suggestive monograph entitled "Demi Fous et Demiresponsibles" (Bibliotheque de philosophie contemporaire), suggests as a partial solution of the problem that institutions or special asylums be established by the state in which it could take proper care of or punish individuals of partially defective mentality, or the half-crazy people, and that the courts should take proper cognisance of this important and numerous class of people and treat them properly. Grassat considers the born criminal of Lombroso as semi-insane. We find in this group of the semi-insane that the saying that one must have a mind before he can lose it is specially true. We find in this group many persons of superior intellectuality who have a high social value. Indeed, some claim that they constitute a majority in this class.

